# Synergistic effects of high fat feeding and apolipoprotein E deletion on enterocytic amyloid-beta abundance

**DOI:** 10.1186/1476-511X-7-15

**Published:** 2008-04-22

**Authors:** Susan Galloway, Menuka MS Pallebage-Gamarallage, Ryusuke Takechi, Le Jian, Russell D Johnsen, Satvinder S Dhaliwal, John CL Mamo

**Affiliations:** 1 School of Public Health and Australian Technology Network (ATN), Centre for Metabolic Fitness, Curtin University of Technology, Perth, Western Australia; 2 Australian Neuromuscular Research Institute, QEII Medical Centre, Perth, Western Australia; 3 Centre for Neuromuscular and Neurological Disorders, University of Western Australia

## Abstract

**Background:**

Amyloid-β (Aβ), a key protein found in amyloid plaques of subjects with Alzheimer's disease is expressed in the absorptive epithelial cells of the small intestine. Ingestion of saturated fat significantly enhances enterocytic Aβ abundance whereas fasting abolishes expression. Apolipoprotein (apo) E has been shown to directly modulate Aβ biogenesis in liver and neuronal cells but it's effect in enterocytes is not known. In addition, apo E modulates villi length, which may indirectly modulate Aβ as a consequence of differences in lipid absorption. This study compared Aβ abundance and villi length in wild-type (WT) and apo E knockout (KO) mice maintained on either a low-fat or high-fat diet. Wild-type C57BL/6J and apo E KO mice were randomised for six-months to a diet containing either 4% (w/w) unsaturated fats, or chow comprising 16% saturated fats and 1% cholesterol. Quantitative immunohistochemistry was used to assess Aβ abundance in small intestinal enterocytes. Apo E KO mice given the low-fat diet had similar enterocytic Aβ abundance compared to WT controls.

**Results:**

The saturated fat diet substantially increased enterocytic Aβ in WT and in apo E KO mice, however the effect was greater in the latter. Villi height was significantly greater in apo E KO mice than for WT controls when given the low-fat diet. However, WT mice had comparable villi length to apo E KO when fed the saturated fat and cholesterol enriched diet. There was no effect of the high-fat diet on villi length in apo E KO mice.

**Conclusion:**

The findings of this study are consistent with the notion that lipid substrate availability modulates enterocytic Aβ. Apo E may influence enterocytic lipid availability by modulating absorptive capacity.

## Background

Net concentration of cerebral Aβ is determined by the presence of apolipoprotein (apo) E with a dose dependent gene effect of apo E -/- < apo E -/+ < apo E +/+ on hippocampal senile plaques [1,2]. Animals and cell culture studies show that apo E regulates the production, transport, clearance and solubility of Aβ [1-8]. Apolipoprotein E may modulate cerebral Aβ homeostasis by regulating cerebral Aβ efflux via the low-density-lipoprotein-receptor-related protein (LRP), relative to the influx of Aβ via transporters such as the receptor for advanced-glycation-end-products (RAGE) [9]. In addition, apo E can also directly influence Aβ biogenesis via regulation of α- and β-secretases activity [10], or indirectly, by influencing the intracellular pool of regulating lipids [11].

Apolipoprotein E critically regulates cholesterol metabolism and lipid homeostasis. The apo E protein is the primary receptor ligand for dietary-derived lipoproteins synthesized by the small intestine (chylomicrons) and triglyceride-rich lipoproteins (very-low-density lipoproteins (VLDL)), synthesized from liver [12]. Several lines of evidence support a link between aberrations in lipid metabolism and AD risk [7,11,13]. Epidemiological and clinical studies suggest that a high intake of saturated fat and/or cholesterol accelerate onset and progression of AD, whereas some polyunsaturated fatty acids may be protective [13-17]. Moreover, strong evidence of a causal relationship between dietary fats and AD comes from feeding studies in mice or rabbits. Animals given saturated-fat diets show significant immuno-detectable cerebral Aβ burden [18-20], although the mechanisms by which this occurs are presently unclear.

Our laboratory recently reported that absorptive epithelial cells of the small intestine secrete Aβ associated with dietary-derived lipoproteins (chylomicrons) [21]. A diet enriched in saturated fats and cholesterol was found to markedly increase enterocytic Aβ, whereas fasting completely abolished Aβ production. Chronic ingestion of saturated-fat may lead to sustained elevations in blood of lipoprotein-bound Aβ, because of overproduction and thereafter, reduced clearance from blood. Moreover, recent studies suggest that exaggerated exposure to circulating Aβ may compromise blood-brain-barrier integrity and exacerbate cerebral amyloidosis [22]. In normal subjects, approximately 60% of lipoprotein-bound plasma Aβ is associated with the triglyceride-rich-lipoproteins (TRL's) and in subjects with AD, post-absorptive accumulation of chylomicrons has been identified [23].

Apolipoprotein E is pivotal for the interaction of TRL with high affinity clearance pathways [12] including the low-density-lipoprotein-receptor (LDL-r) and LRP and will therefore significantly influence plasma lipoprotein-Aβ concentration and kinetics. However, apo E may also influence plasma Aβ homeostasis by modulating synthesis and secretion of the lipoprotein-Aβ complex from either the intestine and/or liver. To explore this concept further, in this study we compared enterocytic Aβ homeostasis in wild-type mice versus animals devoid of apo E (apo E knockouts). Mice were given either a low-fat, or high saturated-fat diet to explore synergistic effects. We find that apo E modulates intestinal morphology in a manner which may influence lipid absorptive capacity and has a synergistic effect with dietary fats on enterocytic Aβ homeostasis.

## Results

### High-fat feeding induced hypercholesterolemia in apo E KO mice

Apo E KO mice given low-fat chow had significantly elevated plasma cholesterol compared to WT mice on the low-fat diet (table 1), however plasma triglycerides were not significantly affected because of the gene deletion. In WT mice the high saturated fat diet had no significant affect on plasma cholesterol or triglycerides (table 1). However, in apo E KO mice hypercholesterolemia was substantially exacerbated and some two-fold greater than the apo E KO mice given low-fat chow. All groups of mice gained weight during the intervention and there was no significant difference between treatment groups (data not shown).

**Table 1:** Plasma lipids in wild-type and apolipoprotein E knockout mice fed low and high fat diets

Diet	Gene	Cholesterol (mM) mean ± S.E.M	Triglyceride (mM) mean ± S.E.M
LF	WT	2.1 ± 0.05	0.69 ± 0.19
LF	Apo E KO	*6.95 ± 1.97	0.68 ± 0.09
HF	WT	2.2 ± 0.46	0.42 ± 0.12
HF	Apo E KO	*14.3 ± 0.01	0.38 ± 0.12

*P < 0.05

S.E.M = standard error of the mean

Table shows plasma cholesterol and triglyceride concentrations (mean ± SEM, n = 6 mice per group) in C57BL/6J WT mice and apo E KO mice maintained on either LF or HF diet for six-months. Apo E KO mice had significantly elevated levels of plasma cholesterol compared to WT controls under both feeding regimens (P < 0.05). High-fat feeding further exacerbated the elevation of cholesterol in apo E KO mice compared to HF-WT (P < 0.001) and LF-APOE KO mice (P < 0.05). Plasma triglyceride was not significantly different between groups.

### Immunolocalisation of Aβ in the small intestine of apolipoprotein E KO mice: synergistic effects of high fat feeding

**Figure 1:**
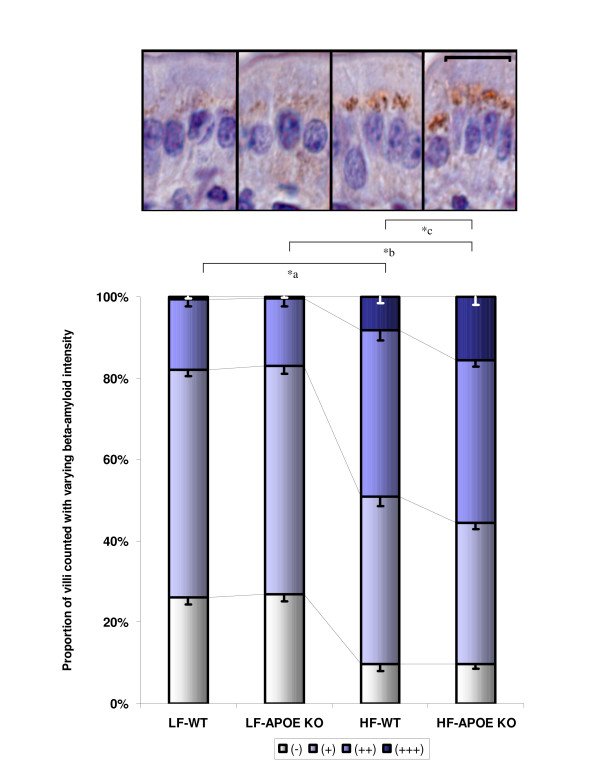
**Enterocytic Aβ in wild-type and apolipoprotein E knockout mice given a high fat diet**. Figure shows proportion of small intestinal epithelial cells with different staining intensity for Aβ. Score as follows: (-) no granular coloration, (+) modest with 1–2 granules, (2+) moderate with 3–4 granules or (3+) high, containing larger intense granules. Data was collected for six mice per group, with a minimum of four tissue sections per mouse studied. A minimum of 200 cells per section were scored and statistical significance was determined by one-way ANOVA with post-hoc Bonferroni test. LF-WT and LF-APOE KO mice have significantly (p < 0.05) fewer cells which stained positive for Aβ compared to mice fed high fats (HF-WT and HF-APOE KO *a and *b respectively). Under high-fat feeding, apo E KO mice had significantly greater proportion of cells which expressed Aβ at higher intensity compared to high-fat fed WT mice (*c, p < 0.05). The inset micrograph shows high-magnification of enterocytes from groups corresponding to graphs below. Beta-amyloid colocalized within the perinuclear regions of the cell containing Golgi and ER within enterocytes from all groups. (Scale bar = 20 μm).

For all groups of mice, Aβ immunostaining was demonstrated within the perinuclear region of absorptive columnar epithelial cells of the small intestine mucosa (insert, figure 1). With low-fat feeding, WT and apo E KO mice exhibited positive staining of Aβ relatively evenly distributed throughout the mucosa epithelium. Apo E KO mice on low-fat diets showed a similar distribution of Aβ compared to WT controls (figure 1). The effect of high-fat feeding on enterocytic Aβ in WT and apo E KO mice is also given in figure 1. Both WT and apo E KO mice had significantly greater enterocytic Aβ abundance, however the effect was more pronounced in the apo E knockout group, notably with more enterocytes showing intense (3+) staining (double asterisks, figure 1).

### Villi height in apo E KO mice and effects of high fat feeding

**Figure 2:**
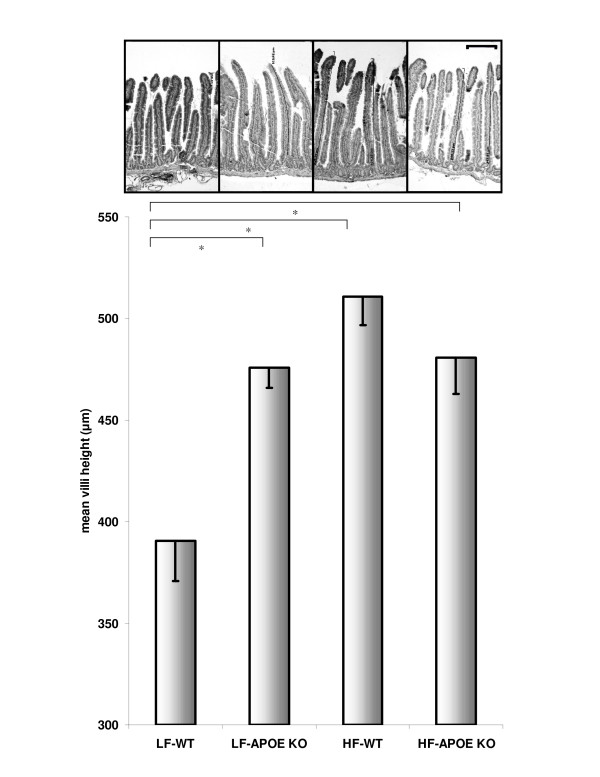
**Villi height in wild-type and apolipoprotein E knockout mice given a high fat diet**. Mean villi height (mm) in WT and apo E KO mice fed low- and high-fat chow. LF-WT group had significantly (*p < 0.05) shorter villi height compared to other groups. The inset micrograph shows low-magnification of intestinal villi height for each group. (Scale bar = 200 μm).

Small intestinal villi length was determined as a surrogate marker of intestinal absorptive capacity. Apo E KO mice on the low-fat diet had significantly greater mean villi length compared to WT controls (figure 2). High-fat feeding was found to substantially increase villi length in control animals and was comparable to apo E KO mice. High-fat feeding had no synergistic influence on villi length in the absence of apo E expression (figure 2).

## Discussion

This study shows that in the absence of apo E, intestinal villi length is significantly greater than WT mice. The absence of apo E coupled with chronic ingestion of a saturated fat and cholesterol diet, increased enterocytic Aβ abundance compared to WT mice on a low-fat diet. This may have simply been a dietary-fat induced effect independent of apo E, because apo E KO mice on a low-fat diet showed similar levels of enterocytic Aβ compared to WT controls. On the other hand, the absence of apo E with a high-fat diet was found to enhance Aβ abundance above that observed in WT mice given saturates and cholesterol. The latter is consistent with modulation of Aβ by apo E that is lipid-threshold dependent.

Apolipoprotein E serves as a TRL ligand for both the LDL receptor and LRP [24,25]. The liver is a major source of apo E, however other tissues including the small intestine express apo E [24,26,27]. Apo E KO mice [28-30] accumulate TRL's because they are unable to bind and be cleared by receptor processes [31]. Under low-fat feeding, apo E KO mice had a greater than three-fold increase in plasma. High-fat feeding exacerbated plasma cholesterol accumulation in apo E KO mice, presumably because of exaggerated lipoprotein production and indeed hypercholesterolemia was increased two-fold above low-fat fed apo E KO mice. Clearance of TRL's from blood is a two-step process requiring triglyceride lipolysis by lipases to produce a depleted apo E rich 'remnant' lipoprotein [24]. Thereafter, remnants are cleared by receptor pathways utilizing apo E as the ligand. There is no hydrolytic defect in apo E KO mice, which explains why these mice were not hypertriglyceridemic.

The mechanisms by which the absence of apo E increased enterocytic Aβ in high-fat fed mice are unclear, although studies in cell culture provide clues. Irizarry *et al *(2004) found that incubation of neuronal cells with apo E resulted in a reduced synthesis of Aβ by lowering the gamma secretase activity [4]. Rough endoplasmic reticulum (rER) and the Golgi compartments are where early endoplasmic cleavage of the Aβ precursor protein occurs, the latter consistent with increased enterocytic perinuclear Aβ immunostaining in apo E KO mice.

This study and others [32,33] found longer villi length in apo E KO mice, suggestive of greater absorptive capacity. Greater substrate availability might stimulate Aβ biogenesis and this hypothesis is supported by the increase in Aβ abundance in high-fat WT mice which also had a marked increase in villus length. Greater Aβ abundance would have been expected in apo E KO mice given the low-fat diet compared to WT controls, because villus length was comparatively greater in the absence of the apo E gene. However, if lipid absorption is already efficient with the low-fat feeding regimen; the deletion of apo E (and increased villus length) would not necessarily have had the expected stimulatory effect on enterocytic Aβ.

Chylomicron synthesis occurs within the ER and Golgi requiring the progressive lipidation of apolipoprotein B_48 _(apo B_48_) [34,35]. Dietary fats transiently stimulate chylomicron synthesis and secretion [36,37] and in clinical studies post-prandial elevations in the Aβ-precursor protein have been reported synergistic with the lipaemic response [3]. How Aβ binds and is secreted with chylomicron is unclear, although the protein is known to bind avidly with negatively charged hydrophobic lipids [5,38]. Cell culture studies also support a lipoprotein mediated secretory pathway because in hepatocyte media, Aβ is found associated with lipoprotein complexes [11].

In animal models and in cell cultures, apo E has confounding effects on hepatic secretion of VLDL. Apo E will normally suppress apo B production, but this is contradicted in the presence of lipids which strongly stimulate lipoprotein biogenesis [39]. In this study, enterocytic Aβ abundance was not significantly different in low-fat apo E KO mice compared to controls, suggesting that chylomicron synthetic rates were not different between these two groups of mice. The increased availability of dietary lipids when animals were fed the high-fat diet would promote chylomicron production and by extension, perhaps Aβ genesis. However, whilst enhanced enterocytic abundance of Aβ was seen in both WT and apo E KO mice given the high-fat diet, the effect was greater in the latter. One explanation is the finding that apo E normally suppresses triglyceride secretion from liver. Therefore, the enhanced effect on enterocytic Aβ seen in apo E KO given high-fat may have been indicative of amplification in the presence of greater cytosolic lipids [39,40].

## Conclusion

Many studies have demonstrated the central role of apo E in maintaining cerebral Aβ homeostasis including modulation of production, as a chaperone protein, and in maintaining efflux and influx pathways across the blood brain barrier. Furthermore, apo E profoundly influences the kinetics in blood of Aβ containing lipoproteins as well as their secretion from liver. This study now demonstrates that apo E may also regulate intestinal Aβ metabolism.

## Materials and methods

### Animals

The protocols described were approved by an ethics committee accredited by the National Health and Medical Research Council of Australia (Curtin University ethics approval N 55-04). Six-week-old female C57BL/6J apolipoprotein E gene knockout (apo E KO) and wild-type (WT) mice weighing approximately 16 g were obtained from the Animal ARC, Perth, Western Australia. Mice were divided and randomly allocated into a low-fat or high-fat diet group. Mice were housed separately in a well-ventilated room that was maintained at 22°C on a 12:12-h light/dark cycles. Body weight was measured weekly.

### Dietary regimen

Chow was purchased from Rodent Diet Specialty Feeds (Glen Forrest, Western Australia). The low-fat (control) group of mice was given chow that contained 4.0% (w/w) as unsaturated fat (AIN93M standard rodent diet) and the diet was free of cholesterol. Mice on the high-fat diet were given chow containing 1.0% (w/w) as unsaturated fat and 16.0% (w/w) as saturated fat (SF00–245 high-fat mouse diet). In addition, the high-fat feed was supplemented with 1% (w/w) cholesterol and 0.5% (w/w) cholate, the latter to aid in absorption. The digestible energy for low-fat and high-fat feed were 15.2 MJ/kg and 18.7 MJ/kg respectively. Food and water were available *ad libitum*.

### Sample collection

After six-months of dietary interventions, mice were anaesthetized with an intraperitoneal injection of Phenobarbital (45 mg/kg). Mice were exsanguinated by cardiac punctureand blood was collected into ethylene-diamine-tetracetic acid (EDTA)-tubes. Plasma was separated by low speed centrifugation and stored at -80°C (under an atmosphere of argon).

### Tissue processing

A small intestine segment measuring 2 cm was cut and isolated from the rest of the digestive tract at the proximal duodenal sphincter. The contents were flushed *in-situ *with phosphate buffered saline (PBS, pH = 7.4), and placed into 10% buffered formalin (ph = 7.4) for fixation. Tissues were fixed for 24 h and processed for immunohistochemistry (IHC).

### Immunohistochemistry

Tissue sections (5 μm) were deparaffinised, rehydrated and IHC analysis was done as previously described [21]. Briefly, the sections were exposed to 3% hydrogen peroxide in methanol for 30 min to quench endogenous peroxidase activity, washed and incubated in blocking serum (20% goat serum) prior to overnight incubation at 4°C with polyclonal rabbit anti-human Aβ_1–40/42 _antiserum (AB5076, Chemicon Temecula, CA), diluted to 1:1000 with 10% goat serum. We previously established specificity by replacing the primary antibody with an irrelevant serum or with PBS and by competition IHC analysis [21]. For the latter, the primary antisera were pre-mixed with solubilised Aβ. Cerebral tissues from transgenic mice (Tg2576sw) expressing familial human APP695 with established plaques were used as positive controls. Slides were washed in PBS and incubated with biotinylated goat anti-rabbit secondary antibody (1:1000 dilution) (E 0432, DAKO, Carpinteria, CA), followed by avidin-biotin-peroxidase complex (ABC/HRP) (K 0377, DAKO, Carpinteria, CA) for 45 min at room temperature. Positive immunostaining was established with liquid diaminobenzidine plus (DAB+) substrate chromogen kit (K 3467, DAKO, Carpinteria, CA). Sections were then counterstained with Harris's haematoxylin.

### Imaging

Digital images for photomicroscopy were acquired by AxioCam HRc camera (Zeiss Germany). Images were captured under identical settings utilising AxioVision software, version 4.5.

### Quantitation of intestinal beta-amyloid abundance

Six animals per group were investigated with a minimum of four tissue blocks prepared for each. From each slide, four images were captured randomly at low magnification (Zeiss AxioVert 200 M, Germany). The intensity of immunolabeling was quantified as previously described [41,42]. Labelling was considered adequate if it was mild (+), moderate (2+), or intense (3+), with adequately labeled positive controls and no labeling in negative controls. The total number of cells with different intensity of Aβ staining was counted by a blinded-to-group investigator in each villus and the data expressed as a percentage.

### Measurements of intestinal villi height

Total of 8 images was taken at low-magnification per group. Representative villi were selected by two independent investigators for height measurement (measurement tool, AxioVision program 4.5).

### Plasma lipid measurements

Plasma lipids were measured immediately following plasma isolation via commercial absorbance-based assays. Triglyceride was determined by measurement of glycerol liberated following enzymatic hydrolysis of triglyceride (TR 1697, Randox laboratories, U.K). Total plasma cholesterol concentration was determined via the cholesterol esterase/cholesterol oxidase technique (CH 201, Randox laboratories, U.K).

### Statistical analysis

The effect of high-fat feeding and apo E gene on Aβ abundance, intestinal villi height, plasma triglyceride and total cholesterol was assessed by univariate analysis. Post-hoc comparisons of means were performed using Bonferroni tests and if equal variance was not found, then Games-Howell test was used to compare difference between individual groups. P-value < 0.05 was considered a statistically significant.

## List of abbreviations

Aβ: beta-amyloid; AD: Alzheimer's disease; Apo: apolipoprotein; APP: amyloid precursor protein; CH: cholesterol; IHC: immunohistochemistry; KO: knockout; TG: triglycerides; TRL: triglyceride-rich-lipoprotein; WT: wild-type.

## Competing interests

The authors declare that they have no competing interests.

## Authors' contributions

SG participated in the design of the study, carried out the study, performed the IHC and lipid analysis and helped to draft the manuscript. MMSPG helped to collect data for results analysis. RT helped to collect data for results analysis. LJ participated in the design of study and performed the statistical analysis. RDJ participated in design of study and helped to draft the manuscript. SSD helped with performing statistical analysis. JCLM participated in the design of the study, performed statistical analysis, and coordinated and helped to draft the manuscript. All authors read and approved the final manuscript.
